# Assessment of immunological response to digital dermatitis pathogen derived antigens following infection, recovery, and reinfection

**DOI:** 10.3389/fvets.2024.1487316

**Published:** 2024-11-28

**Authors:** John W. Coatney, Adam C. Krull, Patrick J. Gorden, Jan Shearer, Samuel Humphrey, Steven Olsen, Paul J. Plummer, Jennifer H. Wilson-Welder

**Affiliations:** ^1^College of Veterinary Medicine, Iowa State University, Ames, IA, United States; ^2^National Animal Disease Center, Agricultural Research Service (USDA), Ames, IA, United States; ^3^United States Department of Agriculture (USDA), Washington, DC, United States; ^4^College of Veterinary Medicine, University of Tennessee, Knoxville, Knoxville, TN, United States

**Keywords:** digital dermatitis, lameness, bovine, animal model, immune response, gamma-delta T-cell

## Abstract

The ability to reliably induce bovine digital dermatitis (DD) in naive calves provides unique opportunities to evaluate immune responses of the calves to infection after disease induction, during healing, and after subsequent re-infection. Dairy calves infected in a previous induction trial were held until lesions resolved and were then re-infected in parallel with naïve calves. Humoral and cell-mediated responses were assessed via serum antibody titer and lymphocyte proliferation analysis with responses of previously infected calves compared with responses of the newly infected calves and naïve calves. In addition, feet of calves in both treatment groups were photographed and scored by a single blinded observer using a previously described induced lesion scoring system. All naïve calves developed lesions after initial infection whereas only 5 of 8 calves developed lesions consistent with DD after a second experimental infection. In the naïve group, lesions commensurate with DD occurred in 15 of 26 experimentally infected feet with 6 feet not included in the analysis due to bandage failure. In comparison, calves in the second infection group developed lesions in 10 of 25 infected feet. Humoral responses or cellular proliferative responses did not differ between the two treatment groups or between calves which developed or did not develop lesions after experimental infection. Our results indicate that resolution of lesions after DD infection, immunity only provides partial protection against reinfection. Further studies are needed to determine immune mechanisms that provide the observed partial protection against reinfection with DD.

## Introduction

Immune responses to digital dermatitis (DD) infection in cattle and specifically, mechanisms for development of protective immunity to DD are poorly understood. Since its initial description as an ulcerative disease of the bovine coronary band in 1974, *Treponema* spp. have been closely associated with the disease in addition to other bacteria, including *Fusobacterium* spp., *Bacteroides* spp., *Porphyromonas* spp., *Campylobacter* spp., and *Dichelobacter nodosus* (1–6). As lesions generally respond positively to topical antimicrobial therapy and shotgun metagenomics has failed to find evidence of viral or fungal DNA; these observations suggest the disease is caused and perpetuated by bacteria (7–12). Recent literature has suggested the etiology of DD is polybacterial with multiple *Treponema* spp. identified as dominant species at various stages of lesion development (13–19). While several treponeme phylotypes are consistently identified in DD lesions, attempts to reproduce the disease using pure cultures of a single species of the cultivable *Treponema* spp. have failed to induce significant lesions (20). A complex (i.e., polybacterial) etiology would suggest that protective immune responses would also be complex, as immunological responses to multiple bacterial species may be required.

Although there are few studies examining the innate and humoral immune responses to *Treponema* spp., little has been done to characterize bovine peripheral memory cellular immune responses to clinical digital dermatitis, especially during acute or chronic disease states, nor have studies examined responses to the multiple proposed polymicrobial etiologies (21–43). Recent success with consistent induction of DD in calves with a macerate from naturally occurring DD lesions provides a model to examine lymphocytic memory responses during DD pathogenesis (44). Based on the lack of published reports on circulating memory and lymphocyte responses to DD, there is a need to characterize these responses in the bovine immune system with known history of digital dermatitis infection, and whether repeated exposure induces protective immunity against re-infection with DD.

Our hypothesis was that development of digital dermatitis, followed by complete recovery, results adaptive immune responses that prevent or decreases the likelihood of subsequent disease after re- infection. We tested this hypothesis by attempting to induce the disease in two groups of calves: one group that had not been previously exposed, and another group in which DD lesions had been successfully induced and fully resolved.

## Materials and methods

### General outline

**Figure 1:**
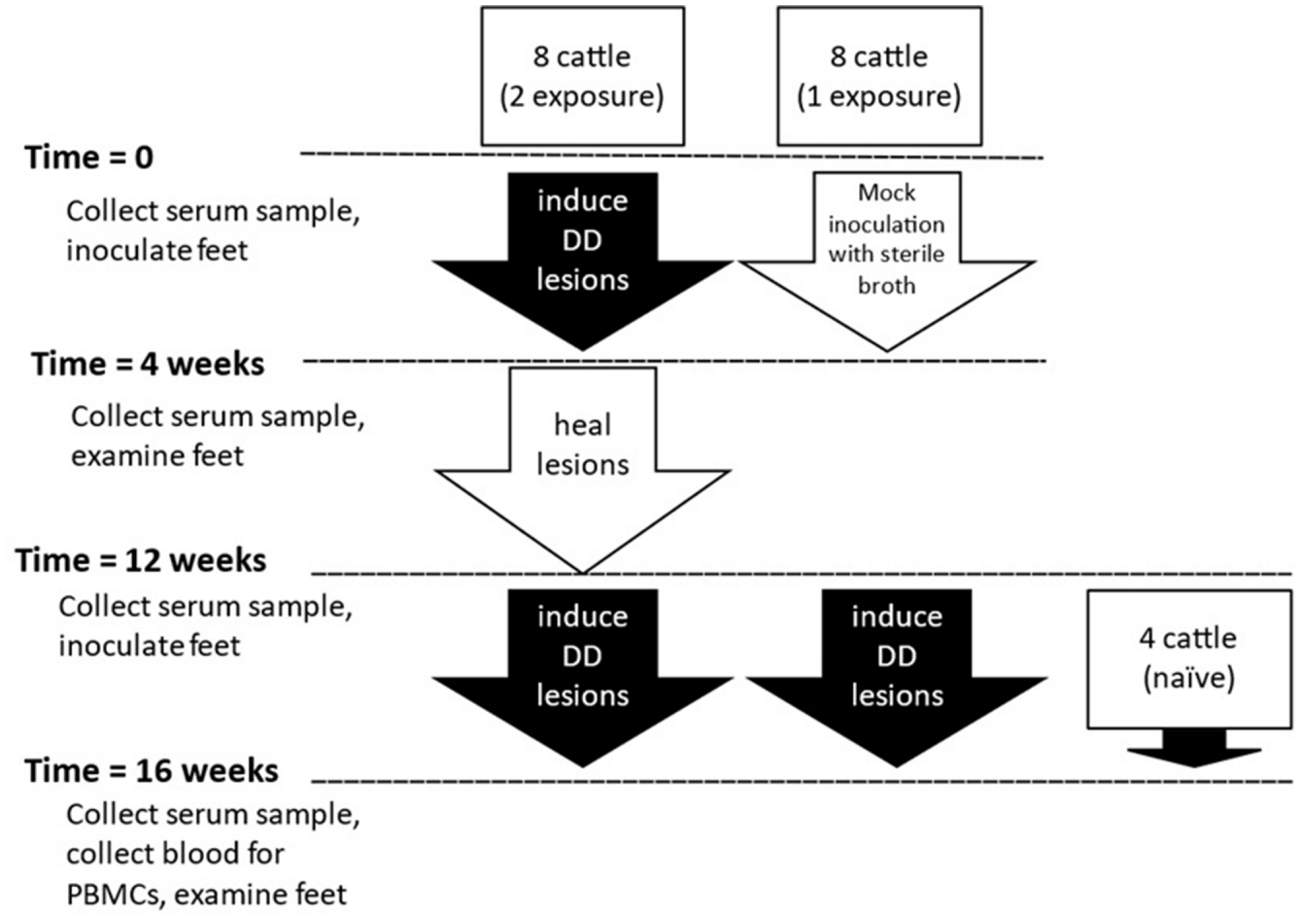
Outline of experimental design.

Holstein dairy calves (*n* = 20) utilized for this study were approximately 250–425 pounds and 4–7 months of age at the beginning of the study. All animal procedures and protocols were approved by the Institutional Animal Care and Use Committee of Iowa State University (IACUC Log #5-14-7795-B) or National Animal Disease Center Institutional Care and Use Committee (number available upon request). Cattle were in three groups based on their previous history, double or two-exposures, single or 1-exposure, or naïve. Schematic of the present study is outlined in Figure 1. Sixteen calves housed at Iowa State University facilities, were taken from a prior study in which digital dermatitis lesions were experimentally induced (44). Because the prior study featured a notably successful novel induction process, these calves in the single induction group (*n* = 8) were negative controls in the previous study, having been “mock” inoculated with only sterile nutrient broth and no DD lesions occurred in any of these animals during the previous study. The two-induction group (*n* = 8) included calves which developed DD lesions after experimental challenge using macerate collected from dairy cows with various stages of digital dermatitis lesions in the previous study. Eleven weeks after the completion of the first induction trial, and after any DD lesions had completely resolved, an experimental challenge was conducted on all four feet of all 16 animals (1- and 2-induction groups) as administered in the previous study. Four Holstein calves of comparable age with a negative history of DD lesions were housed at National Animal Disease Center in isolation facilities that would prevent exposure to the outside environment, including contact with cattle that would transmit digital dermatitis, were included as the naive group.

### Experimental DD lesion induction

The experimental infection was conducted as previously described (44). On Day 0, the skin in the interdigital fold of all four feet were abraded, a 4 × 4 gauze pad soaked in sterile nutrient broth was placed over the site, and heavy-duty duct tape was used to wrap the foot, as previously described (44). On Day 3, inoculum was prepared from biopsy material obtained from DD lesions of various stages in dairy cattle. Biopsy materials were macerated with culture media (Oral Treponeme Enrichment Broth, OTEB, Anaerobe Systems, Morgan Hill, CA) in oxygen-free environment, visually checked to ensure that inoculum contained approximately 1 × 10^7^ spirochetes and deposited underneath wraps in the abraded location using a syringe and teat canula (44). On Day 28, all wraps were removed, and feet were photographed and biopsied. Photographs of lesions were blindly scored by a single observer using an induced lesion scoring system developed for these experiments (44). A score of seven or higher on a 10-point scale was used to indicate a lesion consistent with digital dermatitis. Lesion induction was repeated in both groups of calves following a 4-week healing period.

### Immune response evaluation

Whole cell sonicates preparations of *Treponema denticola, Treponema phagedenis, Treponema pedis, Porphorymonas levii*, and *Fusobacterium necrophorum* were prepared as previously described (42, 45). Blood was obtained for serum preparation from each calf at 0 and 28 days in the first induction trial and at 0, 16 and 28 days in the second trial. ELISA was performed in duplicate using whole cell sonicates characterize humoral response to pathogens that have been proposed as playing a role in the pathogenesis of DD (45). Briefly, bacterial antigens were diluted to diluted to 5 μg/mL (or for *Fusobacterium* to 1 μg/mL) and bound to high binding 96-well titer plates (Costar) incubated overnight. Binding sites were blocked with 5% casein in PBS with 0.05% Tween 20 (PBST). Serum was serially diluted (1:100 to 1:12,800) and incubated for 1 h at 37°C and then overnight at 4°C. Plates were washed 4 times with PBST and 1:25,000 dilution of HRP-conjugated goat anti-bovine IgG (heavy chain) (Bethyl Laboratories Inc., Montgomery TX) was added and incubated for 2 h at 37°C. Plates were washed 4 times with PBST. Hundred microliter KPL Sure Blue Reserve Substrate (SeraCare, Gaithersburg, MD) was added and incubated in the dark for up to 30 min. Reaction was stopped with addition of 100 μL KPL TMB Blue Stop (SeraCare, Gaithersburg, MD) and plates read at 650 nm. Titer is expressed as reciprocal of the highest dilution with optical density 2 standard deviations above average PBS reading. Sixteen weeks after initial inoculation, 50–60 mL of blood was collected in acid-citrate dextrose from each calf. PBMC proliferative responses to bacterial sonicates were characterized under *in vitro* conditions using flow cytometric techniques. PBMCS were isolated from whole blood following established procedures via density gradient centrifugation as previously described (45, 46). Red blood cells were lysed, cells were labeled with Cell Trace Violet Stain (Life Technologies) and cultured at 5 × 10^5^ cells/well in 96 well plates with whole cell sonicates (5 μg/mL) or Concavalin-A (1 μg/μL) in 96 well plates and incubated for 5 days at 39°C and 5% CO_2_. Antibodies for flow cytometry surface staining are listed in Supplementary Table S1 along with a representative gating scheme (Supplementary Figure S1). Following standard conventions, at least 2,000 live lymphocytes identified by viability dye were used for analysis, forward and side scatter profiles were gated for expression of CD4, CD8, γδ-TCR, and CD21. Example of gating strategy is given in Supplementary Figure S1.

### Statistical analysis

Data was analyzed using GraphPad Prism software (version 7) fitting 2-way ANOVA for repeated measures with Tukey’s multiple comparison test for differences within groups between timepoints or within a group across timepoints. ELISA data was log-transformed (Log_2_) before analysis. Differences were considered significant at *p* < 0.05. Since naïve animals were only assayed once, they were excluded from between timepoint analyses.

## Results

### Lesion development

All calves in the single induction group produced DD lesions in at least one foot. Three of the eight calves in the double induction group failed to produce lesions consistent with DD in any feet. When using feet as the unit of measure rather than calves, we found that in the single induction group, 15 of 26 feet developed DD lesions, and six feet were excluded from analysis due to bandage failure during the trial period. In the double induction group, 10 of 25 feet developed lesions, and 7 feet were excluded from analysis due to bandage failure in the induction trial period (Table 1). While results failed to reach statistical significance, a few animals did appear to be protected from lesion development upon second induction (3 of 8 animals).

**Table 1:** Summary of animals and feet in different outcome categories.

	First induction		Second induction		
Group	Number of animals with lesions	Number of feet with lesions^a^	Number of animals with lesions	Number of animals with no lesions	Number of feet with lesions^a^
1x-DD	0 (not infected)	0/32 (not infected)	8^b^	0	15/26^c^
2x-DD	8	32/32	5^b^	0	10/17^c^
2x-DD-P			0^b^	3	0/8^c^

a Animal had to have lesions in all 4 feet to continue to second induction portion of trial. Feet that lost wrap during initial weeks of induction phase were not included in final analysis.

b *P = 0.20.*

c *P = 0.2668, not significant, Fisher’s exact test 90% CI odds ratio.*

### Serum antibody response

**Figure 2:**
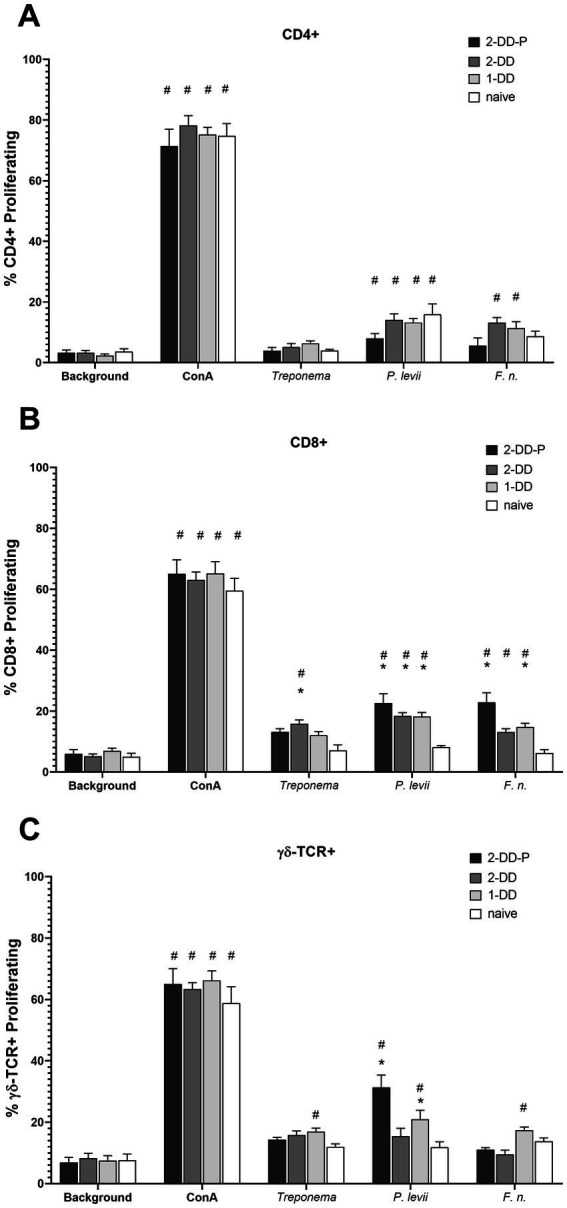
Serum antibody titer measured at 16 weeks or 4 weeks following the second infection. Calves who grossly did not develop lesions or were considered protected (2x-DD-P) were analyzed separate from the calves who were not protected (2x-DD). * Indicates statistical difference from naive within that bacteria (*p* < 0.05), # indicates significance in comparison to background within group (calf treatment), shaded bars depict group means with ± SEM, symbols indicate values for individual animals.

The serum antibody titer to bacteria associated with DD were assessed. Mean serum titers at measured at 16 weeks did not differ (*p* > 0.05) between calves receiving a single DD induction or a second DD induction (double DD) (Figure 2). Furthermore, no differences (p > 0.05) were observed between humoral responses of protected (second induction resulting in no lesions) and unprotected calves (second induction resulting in lesions) (Figure 2). All three DD infection treatments (single-DD, double-DD, double-DD-Protected) were greater (*p* < 0.05) than responses of control/naive calves and the titers to the bacterial lysates increased with exposure (or time) (Supplementary Figure S2) as treatment groups were significantly different from week 0 for most bacterial antigens.

### Cellular response

PBMC proliferating response to bacterial lysates was determined by flow cytometry and phenotype of responsive cells was determined by surface marker antibody labeling. As no differences (*p* > 0.05) in PBMC proliferative responses to *Treopnema* species (*T. denticola*, *T. pedis*, *T. phagedenis*, and *T. vincentii*) were detected, data from these antigen stimulations were combined for further analysis.

**Figure 3:**
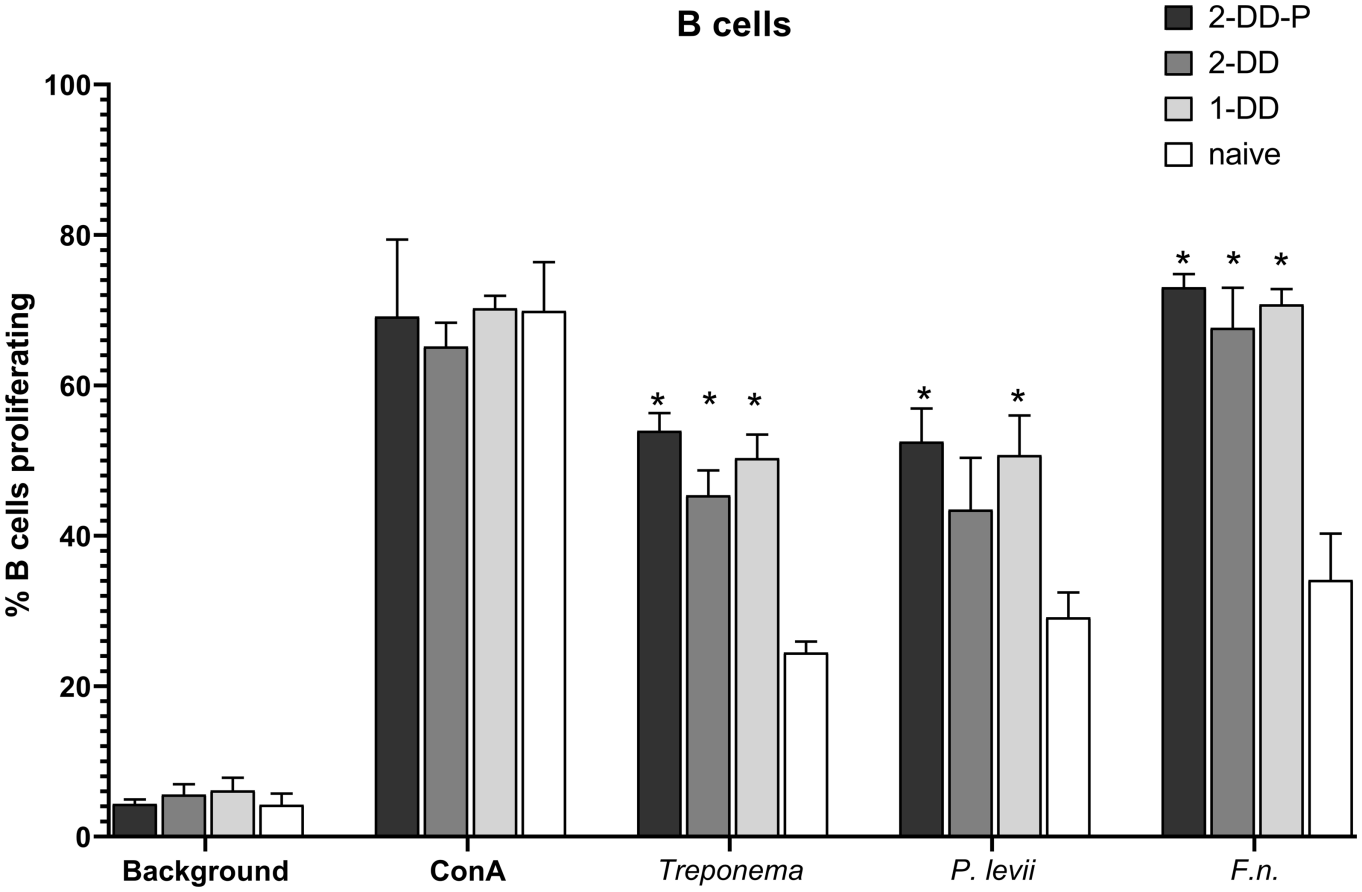
Proliferation of B-cells as identified by CD21 antibody and analysis by flow cytometry. PBMCs were isolated from whole blood and stimulated with ConA (mitogen) or bacterial whole cell antigens for 5 days. Bars represent group means +SEM, * indicates statistical difference from naïve animals for given antigen (*p* ≤ 0.05).

**Figure 4:**
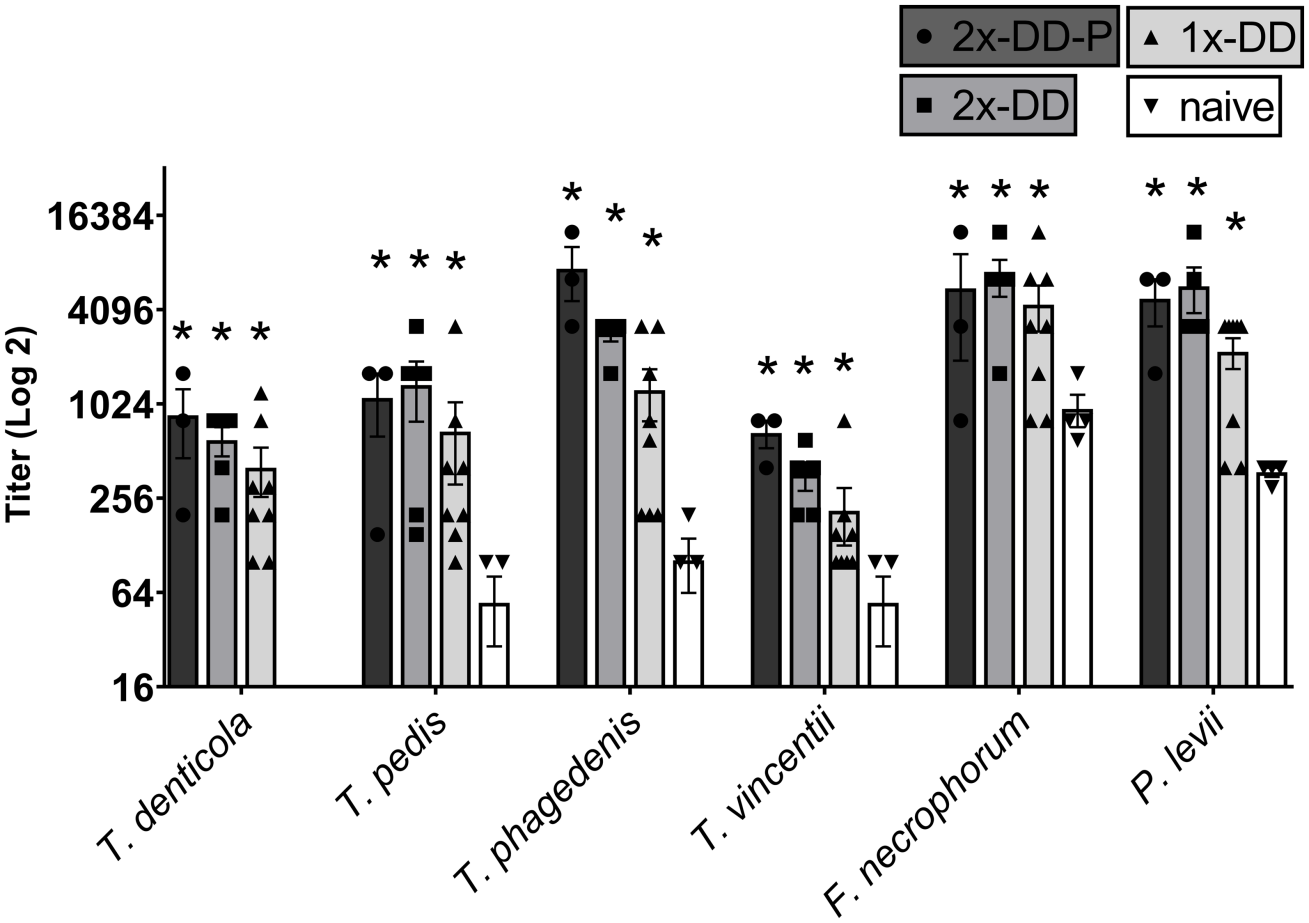
Proliferating lymphocyte response as measured by flow cytometry. Cells were stimulated with media alone (Background), ConA (mitogen) or whole cell sonicates of *Treponema*, *Porphyromonas levii*, or *Fusobacterimum necrphorum* (*F.n*.). (A) Percentage of proliferating CD4+ cells, (B) percentage of proliferating CD8+ cells, (C) percentage of proliferating gamma-delta (γδ) T cells. Bars depict group mean + SEM, * indicates significance between calf group and naïve calves (*p* ≤ 0.05).

In general, B-cells (CD21+ cells) were responsive to all the bacterial lysates, regardless of the infection status of the calves. All calves with experimentally induced DD demonstrated greater (*p* < 0.05) B-cell proliferative responses (Figure 3), especially for *F. necrophorum* antigens. There was no difference between single induction, second induction or second induction-protected (no lesion) responses, only differences between digital dermatitis induced and naïve calves. Proliferation in CD4+, populations to *P. levii* antigens were greater (*p* < 0.05) than background or no stimulation for all calf groups, including naïve, with a strong trend for similar results with the *F. necrophorum* antigen (Figure 4A). Interestingly, the CD4+ response for treponemal antigen was no greater than background or naïve animals, even in the second induction groups, indicating a lack of circulating CD4+ treponemal reactive cells. CD8+ had greater responses (*p* < 0.05) for *P. levii* and *F. necrophorum* and a trend to be higher for all DD induction groups as compared to both naïve and background responses (Figure 4B). Treponemal antigen and *F. necrophorum* reactive γδ + cells were greater than naïve in only the single DD infection treatment, but *P. levii* reactive γδ + cells were greater than naïve calves and background responses for both single induction and the double induction-protected group (Figure 4C). In all cell types, responses to mitogen (ConA) were as expected (e.g., 3–4 times higher than background) and similar across all infection groups.

## Discussion

We postulated that active digital dermatitis would induce protection against reinfection in calves after resolution of initial lesions, theorizing that infection causes significant and measurable immune responses. To address this hypothesis, we induced digital dermatitis in calves that had recovered from experimental DD infections and compared lesions to naïve calves infected in parallel. PBMCs were collected and antigenic responses were compared among the single induction, double induction and unexposed or naïve calves. The number of animals in this study was small and failed to reach statistical significance between protected and unprotected groups, however, there is a trend for protection that is worth following with further research. Indeed, there have been attempts in the past to create a digital dermatitis vaccine, however the bacterins were to only a single organism (13, 27). Numerous studies already mentioned have clearly shown that while *Treponema* spp., may be a key pathogen, there are several species of *Treponema* involved, shifting in dominance as the lesions develop chronicity, along with other key bacterial species. Thus, a single organism bacterin approach will not have much long-term efficacy.

We detected increases in antibody titers to bacterial antigens from *Treponema* spp. and two other pathogens associated with DD, *Fusobacterium necrophorum* and *Porphyromonas levii* (Figure 2; Supplementary Figure S2). Antibody appears to play a minimal role as antibody titers were comparable in the second induction animals, those with or without lesions. Other groups have analyzed the antibody to DD in hopes of developing diagnostic capabilities (5, 21, 26, 28, 39, 47). Differences in antibody titers were noted in some cases but did not correlate with lesion occurrence on an individual (animal level) basis. Others have demonstrated that high antibody titers in cattle correlate with presence of active lesions and increase with severity of lesions, but antibody levels wane as lesions resolve (28, 29, 39, 42). Most of this work has centered around treponemal antigens but increases in antibody responses to other bacteria commonly detected in DD have been noted, including *P. levii* and *F. necrophorum* (33). The pathogenesis of DD suggests that these and other bacterial pathogens (*Dichelobacter nodosus*, *Bacteroides* spp., *Porphyromonas* spp., etc.) may facilitate treponemes establishing colonization in affected tissue. However, at the current time the role of these bacteria in pathogenesis of DD in unknown, as well as the lack of knowledge on bovine immune responses to their co-presence during DD infections.

In this study we also attempted to determine if adaptive immunity might contribute to subsequent protection. While we did induce some degree of protection from re-infection, data did not suggest gross differences in antigen-specific responses in PBMCs. Based on a similar disease, human chronic periodontitis, an increase in CD4+ cells should have been expected (48). Genes within the IL-17 pathway and pro-inflammatory immune response are upregulated in both acute and chronic DD which both increase localized inflammation in the skin via neutrophil recruitment and should result in a Th1/Th17 pro-inflammatory T-cell response (34, 38, 49). One could hypothesize that following active infection, antigen-responsive cells might have localized to either the lymph node or skin rather maintained within circulating populations within blood. While cytokines were not measured in this study, IL-17A can also be produced by bovine CD8+ and γδ T cells (50, 51). Traditionally thought of for viral or intracellular infections, CD8+ T-cells can also play an important role in extracellular bacterial infections, and resident cellular populations in bovine skin (50). This may explain the slight increase in proliferating CD8+ T cells in 2-DD induced groups to treponemal and bacterial antigens when compared to naïve calves. Knowing that CD8 can be co-expressed on γδ T cells, analysis gating of flow cytometry data was conducted so that any CD8+ γδ + cells would be counted as γδ T cells (Supplementary Figure S1). The γδ T cells WC1 receptor is believed to act as a pathogen recognizing bridge between the innate and adaptive immune responses, and are abundant in bovine peripheral blood, peripheral lymph nodes, and skin (52). This cell type contains a transmembrane glycoprotein that serves as a pattern recognition receptor for several specific bacteria, including spirochetes (e.g., *Leptospira*) (53, 54). Trott et al., has demonstrated greater responses in γδ cells in PBMC from natural digital dermatitis-infected cattle when incubated with treponemal antigen (37). Thus, we expected to see more response in the DD induced cattle than we observed. More research on the specific role that γδ T cells may have in the immunological response to digital dermatitis is needed.

Our data suggesting that previous exposure and recovery from DD may provide some protection in cattle against subsequent infection, indicates potential for induction of cellular based immune responses that prevent and/or mitigate the disease. Our data suggests a need for further studies on immunological responses to DD, and potentially other bacterial isolates commonly present in DD lesions.

The most significant limitation in the current study is the difference between an experimental challenge as compared to natural exposure and disease development. In the current study, we have a known clinical history of exposure and high probability of disease development due to experimental challenge conditions. Specifically, experimental conditions such as skin abrasion, high concentrations of inoculum, and maintenance of an environment amenable to infection may have conditions that overwhelmed protective immune responses. Normal skin serves as a natural barrier to prevent exposure to pathogens. It cannot be excluded that greater protection (i.e., lower incidence of lesions in second induction group) would have been observed if inoculation occurred in accordance with exposure under field conditions. However, natural exposure was not feasible for the current experiment. An additional limitation may be the weeks between DD inductions as compared to a production setting, where exposure may be constant or sporadic as animals a moved into and out of facilities or areas, presenting continual exposure to infectious materials. As has been observed with other induction models, a 4-week timeframe is very brief for development of a robust immune response compared to chronicity of natural infection lesions (55–57). Immunological analyses were limited by use of whole cell antigen preparations. Lipopolysaccharide and other antigens can be conserved across Gram-negative bacteria and act as non-specific pathogen-associated cellular activation molecules, however, we were able to include naïve cattle for comparison with each group thus accounting for the non-specific stimulation due to conserved epitopes across bacteria. As more information becomes available on the pathogenesis of DD in cattle, more refined immunologic characterizations may allow detection of mechanisms that contribute to protection or susceptibility of cattle to DD infection.

In conclusion, our data suggests that infection and recovery from DD can provide partial protection against subsequent disease despite lack of evidence for circulating antigen reactive lymphocytes. This suggests that pursuit of a vaccine that prevents or mitigates DD remains a viable possibility worthy of further inquiry.

## Data Availability

The raw data supporting the conclusions of this article will be made available by the authors, without undue reservation.
