# Supplementary figures and images for: Assessment of immunological response to digital dermatitis pathogen derived antigens following infection, recovery, and reinfection
Source: Front Vet Sci. 2024 Nov 28;11:1487316. doi: 10.3389/fvets.2024.1487316 (PMC11660806; doi:10.3389/fvets.2024.1487316)

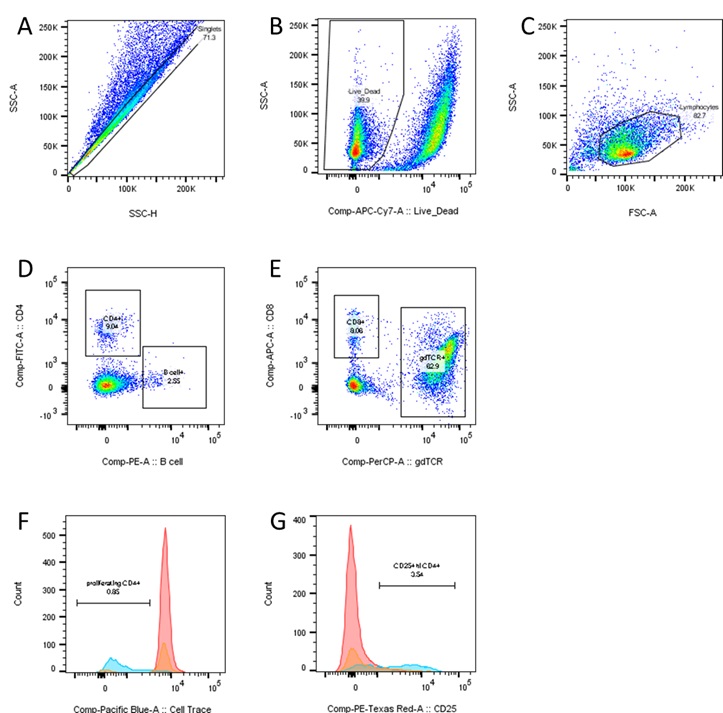

Supplement: Supplementary file 1 [file Image_1.jpg]

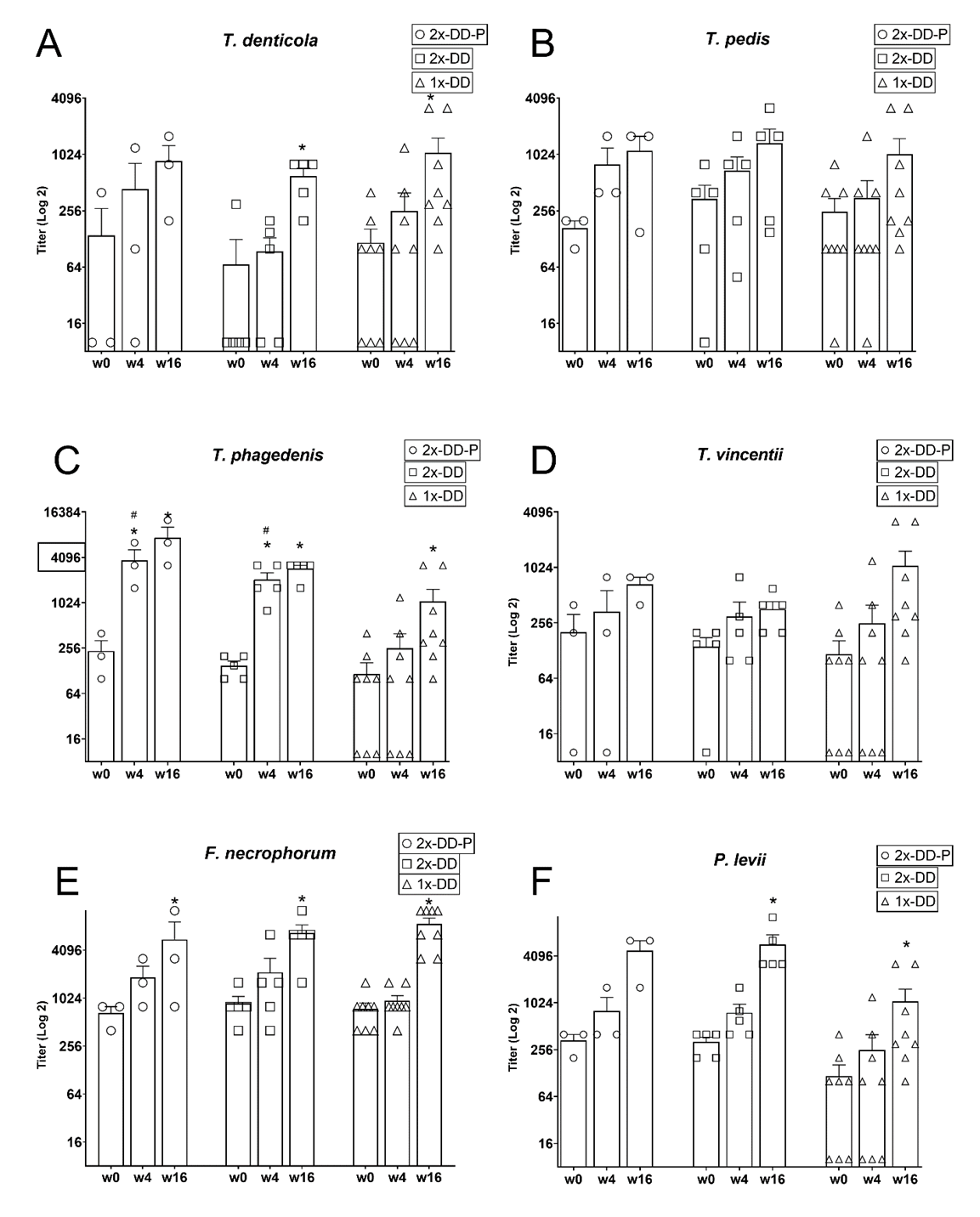

Supplement: Supplementary file 2 [file Image_2.tif]
