# Supplementary material for: Assessment of immunological response to digital dermatitis pathogen derived antigens following infection, recovery, and reinfection
Source: Front Vet Sci. 2024 Nov 28;11:1487316. doi: 10.3389/fvets.2024.1487316 (PMC11660806; doi:10.3389/fvets.2024.1487316)
Supplement: Supplementary file 3 [file Data_Sheet_1.docx]

Supplementary Data:

Table S1: Antibodies and suppliers used for flow cytometry

| Marker | Source | Clone | Secondary | Source |
| --- | --- | --- | --- | --- |
| CD4 | BioRad MCA 1653F | CC8 |  |  |
| CD8 | BioRad MCA 837A647 | CC67 |  |  |
| γδ-TCR | WSU | GB21A | BB700-algG2b | BD Biosciences-745947 |
| CD21 | WSU | Baq15A | PE-aIgM | BioLegend-406508 |
| CD25 | BioRad | IL-A111 | PE TX red-aIgG1 | Southern Bio-0102-13 |
| Live Dead | Thermo-65-0865-14 |  |  |  |

Figure S1: Gating scheme. A. Selection of single cells B. Selection of live cells via dye exclusion (Zombie Yellow). C. Selection of lymphocytes based on forward scatter and side scatter profiles. D. Selection of B cell (CD21) and CD4+ populations. E. Selection of CD8+ and gamma-delta TCR positive populations. Double positive CD8+gdTCR+ were included in gdTCR+ population. F. Proliferating cells selected by diminishing fluorescence of Cell Trace Violet. G. CD25+hi population selection based on histogram. Gate drawn on contrast between background well (red area) and ConA (mitogen) stimulated wells (blue area).

Figure S2: Serum antibody titer to treponeme and anaerobe bacterial whole cell antigens A) *Treponema denticola*, B) *Treponema pedis*, C) *Treponema phagedenis*, D) *Treponema vincentii*, E) *Fusobacterium necrophorum*, and F) *Porphyromonas levii*, determined prior to (week 0) induction, following first induction (week 4) or following the second induction (week 16). Calves that were grossly protected during the second induction (2x-DD-P) were analyzed separately from the calves that developed lesions (2x-DD). * Indicates statistically significant mean within group between timepoints as compared to week 0 (P<0.05). # indicates statistically significant group mean at the given timepoint between calf-induction groups (P<0.05). Bars depict group means +SEM, symbols indicate individual titer values.
